# Strategies for supporting the implementation of a task-shared psychological intervention in South Africa’s chronic disease services: qualitative insights from health managers’ experiences of project MIND

**DOI:** 10.1080/16549716.2022.2123005

**Published:** 2022-09-30

**Authors:** Carrie Brooke-Sumner, Petal Petersen-Williams, Katherine Sorsdahl, James Kruger, Hassan Mahomed, Bronwyn Myers

**Affiliations:** a Alcohol, Tobacco and Other Drug Research Unit, South African Medical Research Council, Cape Town, South Africa; b Alan J Flisher Centre for Public Mental Health, Department of Psychiatry and Mental Health, University of Cape Town, Rondebosch, South Africa; c Department of Psychiatry and Mental Health, University of Cape Town, J-Block, Groote Schuur Hospital, Observatory, Cape Town, South Africa; d Metro Health Services, Western Cape Government: Health, Bellville Health Park, Cape Town, South Africa; e Division of Health Systems and Public Health, Department of Global Health, Faculty of Health Sciences, Stellenbosch University, Cape Town, South Africa; f Curtin enAble Institute, Faculty of Health Sciences, Curtin University, Bentley, WA, Australia

**Keywords:** Psychological interventions, global mental health, implementation strategy, low- and-middle-income countries

## Abstract

**Background:**

Although evidence indicates that task-shared psychological interventions can reduce mental health treatment gaps in resource-constrained settings, systemic barriers have limited their widespread implementation. Evidence on how to sustain and scale such approaches is scant. This study responds to this gap by examining the experiences of South African health managers involved in the implementation of a task-shared counselling service for Project MIND.

**Objectives:**

To qualitatively describe managers’ experiences of implementing the MIND programme and their insights into potential strategies for supporting sustained implementation.

**Methods:**

Two focus group discussions (FGDs) and eight in-depth interviews (IDIs) were conducted with managers of urban and rural primary care facilities in the Western Cape province. All managers were female and 30–50 years old. FGDs and IDIs used an identical semi-structured topic guide to explore the experiences of the MIND programme and perceived barriers to sustained implementation. Normalisation process theory (NPT) guided the thematic analysis.

**Results:**

Four themes emerged that mapped onto the NPT constructs. First, managers noted that their relational work with staff to promote support for the intervention and reduce resistance was key to facilitating implementation. Second, managers emphasised the need for staff reorientation and upskilling to foster openness to mental health practice and for adequate time for quality counselling. Third, managers underscored the importance of strengthening linkages between the health and social service sectors to facilitate delivery of comprehensive mental health services. Finally, managers recommended ongoing monitoring of the service and communication about its impacts as strategies for supporting integration into routine practice.

**Conclusions:**

Findings contribute to the emerging literature on strategies to support implementation of task-shared interventions in low- and middle-income countries. The findings highlight the leadership role of managers in identifying and actioning these strategies. Investing in managers’ capacity to support implementation of psychological interventions is critical for scale-up of these mental health innovations.

## Background

South Africa faces a complex burden of communicable and noncommunicable diseases, including mental disorders [1,2]. Multimorbidity of physical and mental disorders is prevalent, generating complex treatment needs [3]. As in other low- and middle-income countries (LMICs), the treatment gap for common mental disorders (CMDs), such as depression, anxiety and alcohol use disorders, is large [4].

Task-sharing has been recommended as an approach to reducing this treatment gap by facilitating the integration of psychological interventions within primary health care (PHC). In this approach, responsibility for the delivery of psychological interventions is transferred from specialist mental health providers to non-specialist cadres (including facility-based counsellors [FBCs]), with referral to specialist providers if needed. The feasibility, acceptability and effectiveness of task-shared psychological interventions have been evaluated in South Africa [5–10], but these interventions are yet to be delivered as part of routine care due to implementation barriers [11–13]. Implementation strategies (namely systematic processes for introducing and sustaining new practices in routine care) are needed to overcome these barriers [14,15]. In the South African PHC context, health system barriers may include basic challenges of drug stock outs, inadequate infrastructure, long waiting times, and staff shortages [16,17]. Even in high-income countries (HICs), implementing evidence-based psychological interventions at scale is not straightforward, relying on the coordination of a variety of individuals, relationships and processes [18,19]. Information on strategies to support embedding of psychological interventions in routine practice is needed to overcome implementation barriers in South African PHC facilities.

In these constrained PHC facilities, facility and operational managers play a pivotal role in embedding innovations [13]. Their leadership is necessary for building trusting working relationships, influencing organisational climate and ensuring sufficient resources to support implementation [20–23]. These managers are professional nurses who have been promoted to management roles, often with limited management training [24], although many become expert in navigating their work environment to influence change [21,24].

Normalisation process theory (NPT) is one theoretical model that has been used to engage with this complexity and inform strategies to support the integration of new interventions within health services [22,23]. NPT comprises four constructs: *relational integration* refers to the intervention’s fit with the prevailing knowledge and relationships in the setting; *skill set workability* refers to how the intervention affects the current work of staff; *interactional workability* focuses on how the intervention impacts on working relationships and teamwork; and *contextual integration* considers how the innovation fits the organisational context [23].

The current study is nested in the Project MIND trial which investigated two approaches to integrating a new, task-shared counselling service for depression and unhealthy alcohol use into chronic disease care in the Western Cape [25]. FBCs (community health workers employed to provide health promotion and HIV adherence counselling services) were trained to deliver a structured three-session psychological intervention based on motivational interviewing and problem-solving therapy. This intervention is described elsewhere [25–27]. A pre-trial study found substantial variation in facility preparedness to implement this programme, influenced by the leadership style of managers and the overall facility environment [28]. These findings highlight the need for interventions that prepare facilities for the implementation of psychological interventions into PHC facilities. To guide efforts to enhance organisational preparedness for implementation, this study aimed to describe managers’ experiences of implementing the MIND programme and their insights into potential strategies for supporting sustained implementation.

## Methods

This qualitative study is presented according to COREQ guidance [29]. Through this study, we sought feedback from facility or operational managers from the PHC facilities that implemented the MIND counselling programme. The MIND programme was implemented in 16 PHC facilities in the Western Cape. In the parent study [26], 24 PHC facilities were purposively selected as study sites by the Western Cape Department of Health before being randomly allocated to be an intervention site (*n* = 16) or a treatment as usual site (*n* = 8). Convenience sampling was used to obtain the sample for this post-trial study. After obtaining approval from the Provincial Department of Health, the first author contacted the facility and/or operational managers of the 16 intervention sites by email and telephone and invited them to participate in a focus group discussion (FGD). We initially planned to host one FGD per health sub-district, but finding suitable times and arranging these FGDs proved challenging due to conflicting commitments and operational demands of facilities. We managed to conduct two FGDs. The first FGD comprised four managers representing urban facilities and the second FGD involved three managers from rural facilities. Given the difficulty in scheduling these FGDs, we switched to conducting individual in-depth interviews which proved less challenging to arrange. We conducted eight individual in-depth interviews (IDI). One manager declined the offer of an interview due to competing facility demands and their work schedule. At the time of the interviews, this facility had experienced high levels of staff turnover, and the manager was required to provide clinical services in addition to their leadership role.

### Data collection

FGDs and IDIs were conducted by the first and second authors who are experienced qualitative researchers (female, with PhDs). These researchers were known to participants through previous studies but were not linked to the Department of Health. Researchers were familiar with the clinics and the challenges of introducing new services into these contexts, and this may have influenced their interactions with participants. Data collection occurred in a private space at the facility or telephonically. No-one besides the participants and researchers were present during the data collection. FGDs and IDIs were informed by a semi-structured topic guide that explored the overall experiences of the programme and barriers and facilitators to wider implementation (see Supplementary Information for Topic Guide). This topic guide was initially developed by the research team before being presented to our Department of Health partners and stakeholder advisory group for feedback and refinement. Identical questions were asked in the FGDs and IDIs, with the decision to revert to IDIs based on pragmatic reasons. IDIs and FGDs were conducted in English, with some participants responding in Afrikaans. This is common in verbal communication in the Western Cape, where English is the official business language, but many people use both languages in conversation. IDIs and FGDs ranged from 45 to 60 minutes in duration and were audio-recorded before being transcribed verbatim. Audio-recordings were supplemented with fieldnotes. Afrikaans sections were transcribed and translated by a bilingual researcher.

### Data analysis

NVivo 12 software was used to manage the qualitative data. We used the framework approach to analyse data – commonly used by studies with health practice goals and investigating *a priori* factors (in this case, implementation strategies) [30]. The first and second authors initially familiarised themselves with the data before conducting preliminary coding. They then developed a working analytical framework using the four concepts of NPT as *a priori* major themes in relation to the data. They used this framework to independently code five transcripts before meeting to compare codes and revise the framework. The remaining transcripts were then coded according to the revised framework. Cohen’s kappa was calculated for intercoder comparison after coding half of the transcripts. Subsequently, coders (first and second authors) met to discuss coding, focusing on codes for which the kappa indicated less agreement. Coding then continued independently until all the transcripts had been coded, with coders again meeting to compare codes and review coding discrepancies. No new codes or themes emerged after six of the transcripts had been coded, suggesting theoretical saturation [31]. Kappa was calculated with a final value of 0.7, showing good agreement between coders. We held feedback meetings where the results of this and the broader project MIND trial were presented to participating facilities.

## Results

All participants interviewed were female between 30 and 50 years of age. This reflects the gendered composition of the health service. Participant data mapped onto four themes aligned with the components of NPT. These themes are described below with illustrative quotes from participants.

### Theme 1: relational integration – existing knowledge and relationships

Most participants described their facility context as challenging due to diminishing resources and increasing patient numbers. Given this context, participants emphasised the importance of their leadership for ensuring good relationships with staff and facilitating implementation. They recognised their own role in promoting teamwork to support the MIND programme, including onward referral of MIND patients from FBCs for additional specialist care.

*Yes, everybody needs to be together and then when we have something that we have to solve as a group … everybody has a part to play. This morning … I spoke to some of the staff members, we are here 8 hours of the day … so here we need to be like a family. [P.11]*

Participants described the role of managers in introducing and building staff support for the MIND service. For managers, this involved investing time to ‘understand it and internalise’ [P.11] and develop an approach for integrating the new service with current operations. In introducing the new service to staff, participants highlighted the importance of consultation and communicating the added value to patients, providers, and the health system overall. They viewed this aspect of relationship work as critical, given the hierarchical health system where top-down communication and decision-making made staff resistant to new services. When discussing project MIND specifically, participants reflected how they would emphasise the potential for (i) clinical improvements in patients receiving counselling; (ii) improved patient awareness and attitudes to well-being; (iii) improved adherence to chronic medication due to reductions in alcohol use; (iv) better staff understanding of the intersection of social difficulties and health conditions; (v) providing a service to refer patients with complex social and health issues which was previously unavailable; and (vi) up-skilling existing FBCs’ counselling skills. Regular facility meetings were identified as opportunities to present this information, build buy-in and provide feedback on individual patients and the service. One manager described communicating the success of Project MIND to their staff by presenting the positive changes observed because of the programme.

*His [MIND client] whole life was transformed … He would wake up in the morning and drink and he would just sit and his family came in to complain … And I was trying to get them to help him with the ARVs. And they were like “why should we help him, if he is so difficult?” And after Project MIND, this man was like a different person. And he also says he felt like he’s got his life back and he was pleasant. It was like his whole personality changed and his family were all so supportive. That was just one patient. [P.14]*

### Theme 2: skill set workability

Participants described contingencies between the number of patients that nurses screened and referred for MIND counselling and sustained implementation of this service. Participants emphasised the need to improve staff competencies in detecting mental health concerns to ensure sufficient patient referrals. They described how staff avoided asking patients about their mental health because of a lack of knowledge and confidence in this area of practice.

*I think they [nursing staff] are not in tune because mental health has always been a separate service. People are asking to have advanced psychiatry, to manage the mental health patients whereas all of us can do screening. [P.3]*

Participants thought this could be addressed by repositioning patient mental health as everybody’s business and not just the responsibility of psychiatrists. They recommended all facility staff receive a basic orientation to CMDs and the MIND counselling package to enable them to conduct mental health screening and referral.

In addition, participants noted that in some instances, staff roles and responsibilities would need to expand to incorporate mental health care. Managers noted that sustained implementation of the MIND counselling would require FBCs’ job descriptions to be expanded. Managers noted that FBCs had other competing task demands. With this high workload, several participants felt that something would have to *‘*give in the system’ [P.3]. As one participant [P.16] noted,

*If there can be one or two counsellors specifically for [MIND counselling] that will be the ideal thing. That will be the ideal thing because now they have to cut themselves in pieces to cover all their tasks for the day*.

They recommended redefining FBCs’ roles and responsibilities to *‘*create capacity’ [P.1] for selected counsellors to deliver mental health counselling. This would allow them to ‘sit and provide quality’ counselling [P.3]. A related suggestion was to hire additional counsellors to deliver the MIND counselling, but this was considered unfeasible due to resource constraints.

### Theme 3: interactional workability – promoting well-being through onward referral

Onward referral for further mental health and social welfare services is an important aspect of the MIND counselling service requiring interactional workability. FBCs generally referred MIND patients to social workers (where available), and nursing/clinical staff, including mental health nurses within their facility. Participants highlighted the benefit of these referral pathways for improving patient care. Participants felt the MIND programme helped them engage with patients’ underlying problems contributing to their presenting health problem (e.g. interpersonal conflict and violence). This contrasted with the facilities’ usual practice of focusing only on the presenting health concern. Participants highlighted the importance of developing clear referral pathways and processes for successful implementation of the MIND package.

They described several implementation strategies that could support this intervention component. These included physical positioning of FBCs close to social workers or mental health nurses to facilitate ease of referral and warm handover of patients. Others recommended formalising the referral process, using the standard referral letters of the facility. Despite the underlying perception that social workers and mental health nurses are overstretched, participants felt these cadres were glad to receive referrals since the referred individuals were those most in need of additional psychosocial support.

*I think it [Project MIND] was very good, especially for the clients. And for us also because the clients that you [Project MIND] took and referred back to us – those ones we could initiate or change the treatment. [P.12]*

Participants also noted the importance of strengthening referral pathways to external community-based organisations providing mental and substance use disorder treatment. Although participants acknowledged that these services had limited capacity, they highlighted mapping existing services and developing a provider network to whom they could refer patients as important strategies for supporting onward referral. Participants also described existing relationships with several NPOs that could be strengthened to support wider implementation.

*… actually in [area name] there are quite a few NPOs that are working in the community. They have little centres like the Community Centre and there’s another centre near the church where they do rehab. So I think maybe if MIND join up with one of the existing NPOs that’s there. It could actually work. It could be quite successful, especially the ones that are well known. [P.14]*

### Theme 4: contextual integration of counselling

Most participants characterised their facility’s organisational climate as lacking resources, having high caseloads and being in a state of constant policy and practice change. They described difficulties in keeping staff informed about these changes and staff resistance to change. As managers, they felt overstretched and lacking time to address some issues with the attention they would have liked. The need to build resilience among staff, raise morale, and have meaningful engagement with the public around services needs was put forward as key organisational priorities. As one participant noted when asked about the time to implement new initiatives,

*You try - because I change the staff around, so there are other people in the TB room. I can see that it’s getting better but I can’t zoom into it. I think if I get my power in there, there will be a difference, but as I said I really don’t have the time because now it’s stats time again, so I can’t go there … .And then you as a manager need to get the people motivated to take that extra on. [P.11]*

Participants proposed planning, communication and monitoring strategies considered necessary for supporting sustained implementation of MIND counselling within this context. First, participants proposed identifying and prioritising patients most in need of counselling, with appropriate referral for others that the clinic’s counselling service would not have the capacity to serve. It was suggested that patient identification and prioritisation could be achieved by using existing screening guidelines. Second, they offered planning solutions to address facility barriers to counselling uptake that included locating counsellors near the patients’ waiting room, providing counselling in the afternoon when most patients had been seen by nursing staff, and providing convenient appointment times to eligible patients. The lack of private spaces for counselling within facilities was the most frequently described constraint for planning this service. They proposed that strategies for addressing this infrastructure barrier included delivering counselling outdoors when weather permitted, in spaces at nearby community centres and NPOs, or in temporary structures erected on the facility grounds. As one participant suggested

*Maybe you must look at having it in containers on the premises or in one container that you can have as a counselling room. That will solve your problem. [P.13]*

Third, participants emphasised stakeholder engagement and communication as important strategies for supporting implementation. They described how engaging with external stakeholders, including the community served by the facility’s catchment area, would raise awareness of the new service and promote patient uptake of counselling. External stakeholder engagement was also seen as critical to building a network of intersectoral partners to whom patients could be referred should they require additional assistance after receiving MIND counselling. Participants also highlighted the importance of early communication about perceived benefits of the new service with internal stakeholders to build implementation readiness. Managers recommended first communicating with the facility’s department heads who would filter this information to frontline staff. They also proposed having the new service listed as a standing agenda item at the facility’s regular meetings (e.g. staff meetings, morning check-in meetings, and multidisciplinary team (MDT) meetings) to support routinising the intervention into the facility’s communication structures. All participants underscored the importance of engaging with staff about the proposed service prior to providing any training so that any resistance to change could be addressed.

*Staff should also understand that it’s not that we want to give them more work but as I say, as long as it is clarified properly, they should understand. If it is part of the routine care, ideally there should be an agreement between line manager and staff member in terms of expectations. [P.1]*

Furthermore, participants articulated the importance of monitoring the number of patients counselled per month and associated changes in patient health outcomes as a strategy for justifying the continued allocation of resources to the programme. Participants suggested that the MIND counselling programme is included as part of the facility’s annual audit. In this audit, the numbers of patients treated and their health outcomes (for a range of conditions) are assessed against pre-specified targets. As one participant reflected,

*‘I think we would be keeping it on the radar, so it would be good to know what their [FBC] statistics are … . This person has seen this number of clients this month who have completed the modules. … So having it as an expectation that the counsellor see, for example, 5 patients a day and with two counsellors seeing 10 patients a day, in a week 100 patients a week. [P.2]*

## Discussion

Primary healthcare managers are a key lever in the translation of policy to implementation [32], but their views on possible strategies for supporting the sustained implementation of task-shared psychological interventions have rarely been explored [33]. This study begins to address the lack of evidence on implementation strategies necessary for supporting the scale-up of these mental health services in low- and middle-income countries like South Africa.

Although findings indicate broad support for continued implementation of the MIND programme, participants acknowledged systemic challenges to the sustained implementation of this new service. These included infrastructure and workforce barriers as well as contextual factors that influence capability for implementing mental health services more generally (e.g. high workloads and resistance to change). These systemic barriers are consistent with those reported by previous studies [28,33,34].

Using normalisation process theory as a framework, we propose a multicomponent implementation strategy (outlined in Table 1) that reflects managers’ recommendations for overcoming these systemic barriers to sustained implementation of the MIND programme. This strategy includes components targeting service planning and preparation, ongoing communication with internal and external stakeholders, service monitoring, and supervision and support for those delivering the counselling. These approaches align with prior recommendations for supporting the delivery of task-shared interventions in other areas of health care [35].

**Table 1.** Context appropriate implementation strategy for MIND psychological counselling in primary care.

Implementation approach	Facility level	Community level
1. Service planning	Identify key actors: • Counsellors • Heads of departments • Mental health nurses • Social workers	Map community actors: • Health committee • NPOs and/or religious organisations • Department of Social Development (social workers)
	Consult with key actors on: • Space for counselling • Appointment times	Communicate with key actors on: • Possible referrals • Community venues for counselling
2. Orientation and training	Subdistrict facility manager meetings • Challenges and approaches for this new service	Health Committee Meeting • Introduction to the service • Introduction to counsellors • Printed/social media introducing service
	Staff meeting: • Introduction to service • Mental health and wellness check-in with staff • Mental health reorientation for all staff	
	Heads of department meeting: • Present benefits of programme • Present roles and responsibilities, referral pathways	
	Training workshops: • Counselling skills training for counsellors, mental health nurses, and social workers (doctors and other nurses)	
	Introducing service to community: • Waiting room talks • Printed and social media	
3. Monitoring	Monitoring meetings • Clinical and MDT meetings • Feedback and follow-up as needed	Health Committee Meetings • Standing agenda point • Feedback to facility manager • Referrals to NPOs and feedback • Patient feedback through suggestions box and to health committee
	Staff meetings • Standing agenda point for feedback and discussion on programme	
	Monitoring statistics and reporting • Targets for counselling sessions • Feeding into monthly and annual reports (facility and NPO)	

Our findings highlight activities needed to support *contextual integration* of the intervention. According to managers, these include the appropriate introduction of staff to the ‘new’ work in a way that would minimise resistance. Managers also described the need for a cycle of communication and feedback between counsellors, clinicians, and implementation planners. This type of ‘responsive feedback’ is vital for enabling a service to respond to complex and changing contexts that affect intervention delivery and can keep the service on course to attain desired outcomes [36].

In terms of *relational integration*, our findings underscore the role of managers in promoting teamwork and good working relationships amongst staff which they considered essential to securing support for implementation of the new service. Managers in this study described how they used their leadership position to synthesise information about the intervention to make it relevant to staff and justify the service change. They also described their role in reframing challenges to implementation (e.g. through presenting the benefits of the innovation) and in strengthening social networks within the organisation and the wider community as described by other studies [20,37]. Managers reflected how these strategies increased resilience to service changes amongst staff and promoted positive engagement with the intervention amongst both staff and the broader patient community.

For the *skill set workability* construct, managers underscored perceptions amongst staff that all mental health conditions should be treated by a mental health specialist. They described primary care staff as lacking skills and confidence to screening and treating patients for CMDs, even though current primary care guidelines specify these should be addressed in primary care [38]. Constrained resources, limited mental health training, and lack of support for staff likely contribute to staff reluctance to screen patients for mental health concerns [28,33,39]. Part of the suggested implementation approach (Table 1) includes providing mental health orientation training to primary care facilities to build capacity for the delivery of mental health services on the primary care platform. Other strategies to build the skill sets required include discussing the programme at regular facility meetings (as outlined in Table 1) to build working relationships and foster interprofessional collaboration in the delivery of mental health services.

In relation to *interactional workability*, managers stressed the need for pathways through which counsellors could refer patients to more specialised services if they required additional support, highlighting the need to develop these referral pathways during preparation for implementation (Table 1). The upward referral of patients to more specialised services is an integral aspect of effective task sharing for mental health care [40] but remains challenging in South Africa due to inadequate investment in human resources for mental health (including mental health nurse specialists) [41,42]. Addressing the chronic shortage of mental health specialists in this setting will be key to supporting the implementation of task-shared mental health services. Furthermore, participants described the scarcity of facility-based social workers as another challenge to the provision of comprehensive mental health services. Lack of access to social services is a key barrier to addressing substance use and gender-based violence that often co-occur with mental health problems [43,44]. Consequently, participants highlighted the need to strengthen outward referral pathways to NPOs that provide these social services. The importance of strengthening linkages between the health and social service sectors as a strategy for supporting the mental health care of patients has been noted by previous studies [13,34,41].

Finally, it is highly likely that managers will require support to implement the MIND programme. This implementation support is often lacking in the South African health system where plans and policies are often filtered to the primary level, without accompanying guidance on implementation [13]. Applying the implementation strategy described in Table 1 may support managers to implement this and other innovations. Leadership development interventions for managers that include peer mentoring and support, opportunities for sharing of experiences and reflection on practice have been proposed as a means of building capacity among health managers for the introduction and sustained implementation of new evidence-based practices [25,45]. A variety of these interventions exist, including the Leadership and Organizational Change Intervention [46], but these would require contextual adaptation to ensure appropriateness for the South African context.

### Methodological considerations

First, we relied on managers’ views of implementation barriers; frontline staff may have had alternative or additional insights into how to facilitate implementation of the MIND programme. Second, one of the two interviewers had been involved in the trial, and this may have introduced some socially desirable responses. We tried to minimise this by reassuring participants that we were seeking constructive feedback about how to better support future implementation of this programme. Third, we acknowledge that the combined use of interviews and focus groups is a limitation, as the data generated by each method is fundamentally different. Fourth, while we did present the findings back to participants in facility-wide feedback meetings, transcripts were not returned to participants and member checking did not form part of the data analysis process. Future studies should strive to include this to increase confidence in the trustworthiness of the results. Fifth, our sample was limited even though participants represented most of the MIND intervention sites. We do not know the extent to which this sample was representative of the 189 facilities in the province. Furthermore, facilities in the Western Cape may be better resourced for health services compared with other provinces, potentially limiting the applicability of implementation strategies in facilities with differing organisational climates and community contexts. Finally, while the results of this study support the key constructs outlined in Normalisation Process Theory, our analysis did not reveal information that could be used to further develop the framework. Future studies that examine the utility of this framework could help support implementers of mental health interventions in PHC services.

## Conclusion

This paper presented PHC managers’ experiences of implementing a task-shared psychological intervention and their insights into strategies for overcoming systemic and contextual barriers to sustained implementation. These findings contribute to the emerging literature on implementation strategies for mental health innovations in low- and middle-income countries. Using normalisation process theory as a conceptual framework, we used the study findings to develop a multicomponent strategy for supporting sustained implementation of the MIND programme. The study highlights the leadership role that managers play in actioning these strategies. Investing in managers’ capacity to support implementation of mental health innovations is therefore critical for interventions aimed at reducing the mental health treatment gap in resource-constrained settings to realise their promise.

## Paper context

This study presents health care managers’ insights into strategies for overcoming barriers to implementation of a task-shared psychological intervention within South African primary care facilities. These insights were used to develop a multicomponent strategy for supporting sustained implementation of the service. Findings contribute to the emerging literature on implementation strategies for mental health innovations in low- and middle-income countries. Findings may help others grappling with how to support implementation of evidence-based interventions in these settings.
